# ^18^F-FDG PET baseline radiomics features improve the prediction of treatment outcome in diffuse large B-cell lymphoma

**DOI:** 10.1007/s00259-021-05480-3

**Published:** 2021-08-18

**Authors:** Jakoba J. Eertink, Tim van de Brug, Sanne E. Wiegers, Gerben J. C. Zwezerijnen, Elisabeth A. G. Pfaehler, Pieternella J. Lugtenburg, Bronno van der Holt, Henrica C. W. de Vet, Otto S. Hoekstra, Ronald Boellaard, Josée M. Zijlstra

**Affiliations:** 1 Cancer Center Amsterdam, Department of Hematology, Amsterdam UMC, Vrije Universiteit Amsterdam, De Boelelaan 1117, 1081 HV Amsterdam, Netherlands; 2 Department of Epidemiology and Data Science, Amsterdam UMC, Vrije Universiteit Amsterdam, Amsterdam Public Health Research Institute, De Boelelaan 1117, Amsterdam, Netherlands; 3 Cancer Center Amsterdam, Department of Radiology and Nuclear Medicine, Amsterdam UMC, Vrije Universiteit Amsterdam, De Boelelaan 1117, Amsterdam, Netherlands; 4 Department of Nuclear Medicine and Molecular Imaging, University of Groningen, University Medical Center Groningen, Groningen, The Netherlands; 5 Department of Hematology, Erasmus MC Cancer Institute, Wytemaweg 80, 3015 CN Rotterdam, The Netherlands; 6 Department of Hematology, HOVON Data Center, Erasmus MC Cancer Institute, Dr. Molewaterplein 40, 3015 GD Rotterdam, The Netherlands

**Keywords:** Diffuse large B-cell lymphoma, Prediction, Radiomics, ^18^F FDG PET/CT

## Abstract

**Purpose:**

Accurate prognostic markers are urgently needed to identify diffuse large B-Cell lymphoma (DLBCL) patients at high risk of progression or relapse. Our purpose was to investigate the potential added value of baseline radiomics features to the international prognostic index (IPI) in predicting outcome after first-line treatment.

**Methods:**

Three hundred seventeen newly diagnosed DLBCL patients were included. Lesions were delineated using a semi-automated segmentation method (standardized uptake value ≥ 4.0), and 490 radiomics features were extracted. We used logistic regression with backward feature selection to predict 2-year time to progression (TTP). The area under the curve (AUC) of the receiver operator characteristic curve was calculated to assess model performance. High-risk groups were defined based on prevalence of events; diagnostic performance was assessed using positive and negative predictive values.

**Results:**

The IPI model yielded an AUC of 0.68. The optimal radiomics model comprised the natural logarithms of metabolic tumor volume (MTV) and of SUV_peak_ and the maximal distance between the largest lesion and any other lesion (Dmax_bulk_, AUC 0.76). Combining radiomics and clinical features showed that a combination of tumor- (MTV, SUV_peak_ and Dmax_bulk_) and patient-related parameters (WHO performance status and age > 60 years) performed best (AUC 0.79). Adding radiomics features to clinical predictors increased PPV with 15%, with more accurate selection of high-risk patients compared to the IPI model (progression at 2-year TTP, 44% vs 28%, respectively).

**Conclusion:**

Prediction models using baseline radiomics combined with currently used clinical predictors identify patients at risk of relapse at baseline and significantly improved model performance.

**Trial registration number and date:**

EudraCT: 2006–005,174-42, 01–08-2008.

**Supplementary Information:**

The online version contains supplementary material available at 10.1007/s00259-021-05480-3.

## Introduction

Diffuse large B-cell lymphoma (DLBCL) is the most common subtype of aggressive non-Hodgkin lymphoma (NHL) in adults. Up to one third of these patients fail to achieve complete remission during first-line treatment or experience relapse, and salvage treatment regimens lead to modest cure rates [1, 2]. Identification of high-risk patients with the current prognostic scoring systems, such as the international prognostic index (IPI), is limited [3, 4]. Therefore, more accurate prognostic markers are essential to identify patients at high risk for progression or relapse. These poor responders might benefit from an early switch to novel therapies aiming to improve outcome.

Quantitative ^18^F-fluorodeoxyglucose positron emission tomography (^18^F-FDG PET) parameters, especially baseline metabolic tumor volume (MTV), have shown to be predictive of outcome in DLBCL [5–9]. MTV reflects the ^18^F-FDG-avid tumor burden, but it does not comprise phenotypical aspects like spatial distribution, heterogeneity, and shape of lesions. Recently developed quantitative ^18^F-FDG PET image features, also referred to as radiomics, reveal biological characteristics of disease and could help to improve outcome prediction in DLBCL at baseline. Radiomics features capture detailed and quantitative information on, e.g., texture and shape of lesions. In several solid tumors, radiomics features provide prognostically relevant information [10–13]. Evidence is emerging to suggest that such parameters may also have predictive value in DLBCL [14, 15]. However, these parameters have not yet been successfully integrated with IPI components. The objective of this study was to assess the added value of baseline quantitative radiomics features in DLBCL patients compared the currently used IPI score. Secondary objectives were to assess the added value of radiomics to other clinical characteristics and MTV.

## Methods

### Study population

Newly diagnosed DLBCL patients from the multicenter randomized phase 3 HOVON-84 trial (EudraCT, 2006–005,174-42) who underwent baseline ^18^F-FDG PET/computed tomography (CT, ^18^F-FDG PET/CT) were included in this study. ^18^F-FDG PET/CT scans were included from 58 different hospitals. Main inclusion and exclusion criteria of the trial have been published elsewhere [16]. As there was no difference in time to progression (TTP) between the two treatment arms, all available data for this study was used (Supplemental Fig. 1). The HOVON-84 study was approved by the institutional review board (Erasmus MC, 2007–055), and all participants gave written informed consent to participate.

### Quality control of ^18^F-FDG PET/CT scans

Baseline ^18^F-FDG PET/CT scans were centrally collected from participating sites in DICOM format and de-identified. For quality control (QC), we used criteria described by EANM guidelines: mean standardized uptake value (SUV_mean_) of the liver should be between 1.3 and 3.0 and the plasma glucose lower than 11 mmol/L [17]. QC rejected scans if (1) scans were not complete, (2) essential DICOM data was missing, (3) the liver SUV_mean_ was outside the acceptable ranges, and the total image activity (MBq) was not between 50 and 80% of the total injected FDG activity or (4) plasma glucose exceeded 11 mmol/L.

### Quantitative image analysis

Quantitative PET/CT analysis was performed using the ACCURATE tool [18]. Lesions were delineated using a fully automated preselection of ^18^F-FDG-avid structures defined by a SUV ≥ 4.0 and a volume threshold of ≥ 3 mL. Non-tumor regions were deleted, and lymphoma lesions < 3 mL were added with single mouse clicks. If tumor regions were adjacent to non-tumor ^18^F-FDG-avid regions (e.g., kidney, bladder), non-tumor regions were removed manually. Details on the delineation methods and workflow are described elsewhere [19, 20]. All scans were reviewed by a nuclear medicine physician, and delineations were performed under supervision of a nuclear medicine physician.

### Feature extraction

Four hundred eighty features pertaining to morphology (n = 22), intensity (n = 50), and texture (n = 408) (Supplemental data) were extracted both for the individual lesions as for the complete MTV (patient level). Before feature calculation, all images were resampled to 2 × 2 × 2 mm voxel size using tri-linear interpolation. In order to calculate textural features, the images were discretized with a fixed bin size of 0.25 SUV [21]. Furthermore, 5 conventional PET features were extracted from the original images (without resampling): MTV, SUV_max_, SUV_peak_, SUV_mean_, and total lesion glycolysis (Supplemental data). All image processing and feature calculations were performed using RaCat software [22], which is in compliance with the Image Biomarker Standardization Initiative (IBSI) [23].

The patient level VOI included all segmented lesions and was generated by assigning all voxels within the individual lesions to one and all voxels outside any of the segmented individual lesions to zero. At patient level, 5 conventional PET features and 5 dissemination features were extracted: the number of lesions and 4 features as suggested by Cottereau et al. [15], the distance between the 2 lesions that were furthest apart (Dmax_patient_), the distance between the largest lesion and the lesion furthest from that bulk (Dmax_bulk_), the sum of the distances from the largest lesion to all other lesions (spread_bulk_), and the sum of the distances from all lesions to all the other lesions (spread_patient_). Distances were calculated based on the location of the SUV_max_ for each lesion.

### Clinical predictors

For the currently used clinical predictors, the IPI score [24], the individual components of the IPI score (Ann Arbor stage, lactate dehydrogenase (LDH) level, extranodal (EN) involvement, WHO performance status, and age), and bulky disease (diameter lesion ≥ 10 cm) were used. For the clinical predictors, Ann Arbor stage was included both dichotomously and categorically. LDH was included both dichotomously and continuously, for which the LDH level was divided by the upper limit of normal (ULN). EN involvement and WHO performance status were used with two different cut-offs (EN involvement, ≥ 1 or > 1; WHO performance status, ≥ 1 or ≥ 2). For two patients, WHO performance status was missing; these values were imputed as WHO performance status 0 for the IPI score. For the IPI prediction model, patients were divided into four prognostic IPI subgroups (low, low-intermediate, high-intermediate, and high) [24].

### Statistical analysis

The primary endpoint was 2-year time to progression (TTP), defined as time from baseline PET/CT to progression. Patients who died without progression were censored at date of death. Patients still alive were censored at date of last contact.

The predictive value of the following models was assessed:

- Model 1. IPI
- Model 2. Clinical model
- Model 3. MTV at patient level
- Model 4. Limited radiomics model: conventional PET, dissemination, and sphericity features (e.g., commonly used radiomics features) at patient level
- Model 5. All radiomics features for the largest and hottest lesions, respectively
- Model 6. Combination of the clinical predictors (model 2) and radiomics features (model 4) (Table 1)

**Table 1:** Description of prediction models included in this study

Models	Included features
Model 1: IPI	IPI
Model 2: clinical model	Ann Arbor stage, age, WHO performance status, extranodal involvement, LDH, and bulky disease
Model 3: MTV	MTV
Model 4: limited radiomics model	MTV, SUV_max_, SUV_peak_, SUV_mean_, TLG, number of lesions, Dmax_patient_, Dmax_bulk_, Spread_patient_, Spread_bulk_, and Sphericity
Model 5: all radiomics features (largest and hottest lesion)	485 features for the largest and hottest lesion
Model 6: combined model	Features model 2 and model 4

Abbreviations: *IPI,* international prognostic index; *LDH,* lactate dehydrogenase; *WHO,* World Health Organization; *MTV,* metabolic tumor volume; *SUV,* standardized uptake value. *TLG,* total lesion glycolysis; *Dmax,* maximum distance

To evaluate model performance for 2-year TTP, the receiver operator characteristic curve was generated to calculate the area under the curve (AUC). A 95% confidence interval (CI) of the AUC and differences between model performances of prediction models, expressed as AUC, were assessed with the two-sided DeLong test [25]. Stratified repeated cross-validation with fivefold and 2000 repeats was performed to yield the cross-validated AUC (CV-AUC). High- and low-risk groups were defined based on prevalence [26] as follows: in our dataset, 52 patients had an event at 2-year TTP. For the IPI prediction model, patients with 4 or 5 adverse factors were considered as high risk. For the multivariate models, the high-risk group was defined as the 52 patients who had the highest predicted risk of progression (Supplemental data). Diagnostic performance was assessed using sensitivity, specificity, positive predictive value (PPV) and negative predictive value (NPV), and log-likelihood ratios. Patients censored before 2 years of follow-up were excluded for the prediction models and diagnostic performance. To assess the robustness of our model predictions, a sensitivity analysis with 2-year progression-free survival (PFS) as outcome parameter was performed for all prediction models.

For all models except model 5, multivariate logistic regression with backward selection was used to predict outcome. For models 4 and 6, to reduce the radiomics feature space dimension, the previously reported features regarding intensity, volume, shape, and dissemination of the lesions were preselected (Supplemental data). For model 5, LASSO logistic regression was performed after mean centering and scaling by standard deviation of all features. Prior to analysis, continuous input variables that had a skewness > 0.5 were log-transformed using the natural logarithm. To compare model performance of models 1–4 and 6 to the model performance of model 5, we also used LASSO logistic regression to predict outcome for these models.

Survival curves were obtained with Kaplan–Meier (KM) analyses for TTP and compared with log-rank tests for the IPI, best clinical, MTV, best radiomics, and best combined prediction models based on logistic regression. In our dataset, 16% of all patients had progression at 2-year TTP, so for each model, 16% of the patients with the highest risk were included in the high-risk group for all KM survival plots except for the IPI KM survival plot, for which we used the high-risk IPI group as high-risk group. Univariate Cox regression models were used to calculate hazard ratio’s (HR) and their corresponding 95% confidence intervals. The assumption of proportional hazards was assessed based on Schoenfeld residuals.

Statistical analysis was performed using R (version 4.0.0). A *p* value of less than 0.05 was considered statistically significant.

## Results

### Patient characteristics

Three hundred seventy-three patients had a baseline PET/CT, of which 317 were included in this analysis. The main reason for ineligibility was missing essential DICOM information (n = 21). Other reasons for exclusion were QC outside of range (n = 19), incomplete whole-body or total-body PET/CT scans (n = 13), no FDG-avid lesions (n = 2), and plasma glucose out of range (n = 1). Clinical characteristics of included patients are summarized in Table 2. Fourteen patients (median age, 73; range 53–79) died without signs of progression before 24 months (n = 6 complications of treatment, n = 2 s malignancy, n = 2 intercurrent disease, n = 2 other reasons, n = 1 unknown, and n = 1 non-Hodgkin lymphoma), and 7 patients were lost to follow-up within 24 months, leading to exclusion for the prediction model.

**Table 2:** Patient characteristics

	N (%)
Age	
Median (range) ≤ 60 years > 60 years	65 (23–80) 102 (32) 215 (68)
Sex	
Male Female	161 (51) 156 (49)
Ann Arbor Stage	
2 3 4	51 (16) 66 (21) 200 (63)
LDH	
Normal > Normal	104 (33) 213 (67)
Extranodal localizations	
≤ 1 > 1	186 (59) 131 (41)
WHO performance status	
0 1 2 missing	179 (56) 97 (31) 39 (12) 2 (1)
IPI	
Low Low-intermediate High-intermediate High	52 (16) 77 (24) 109 (34) 79 (25)

Abbreviations: *LDH*, lactate dehydrogenase; *WHO*, World Health Organization; *IPI*, international prognostic index

### MTV analysis

**Fig. 1:**
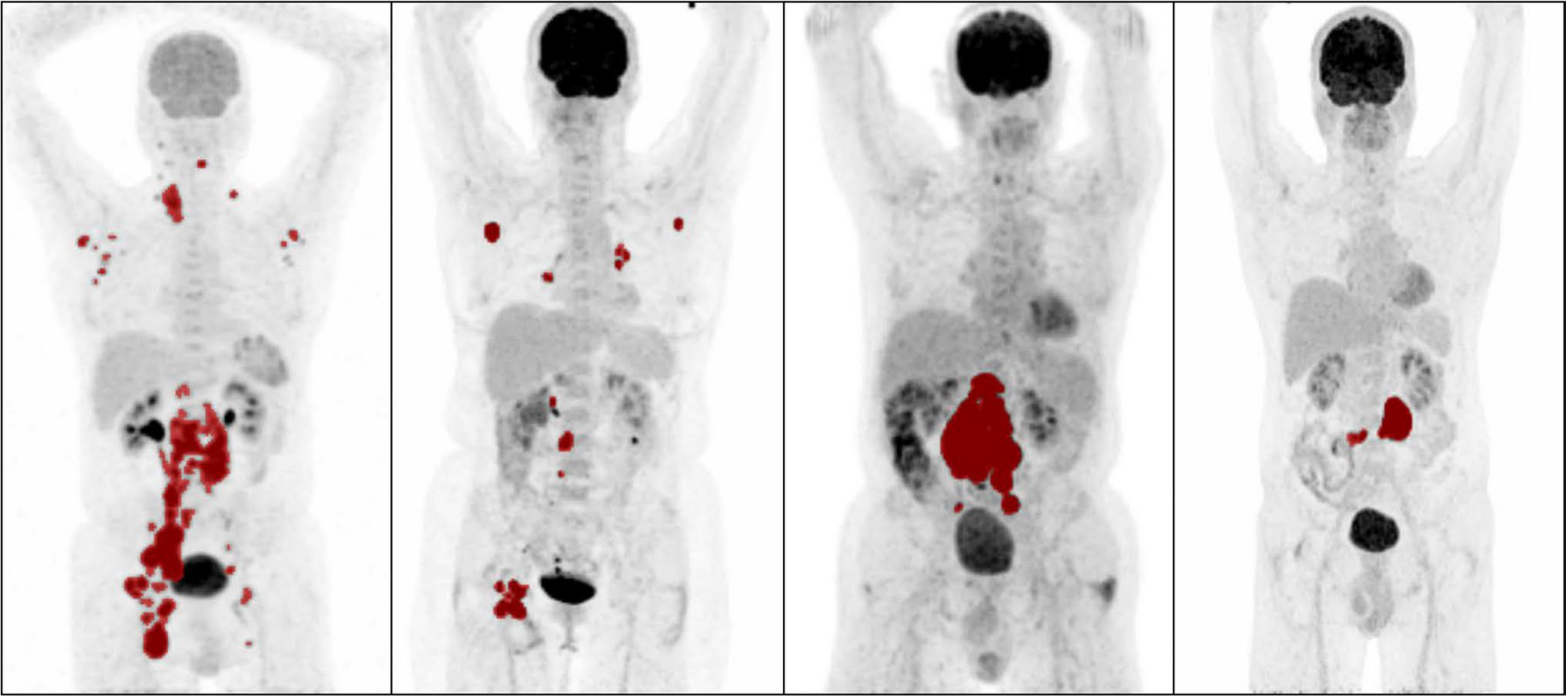
Maximum intensity projections of patients with high MTV, low MTV, high dissemination, and low dissemination. Tumor delineations are indicated in red. From left to right: high MTV and high dissemination, low MTV and high dissemination, high MTV and low dissemination, and low MTV and low dissemination

Per patient, 1–143 lesions were analyzed, with a median of 19 lesions per patient for patients who experienced relapse or progression within 2 years and a median of 8 lesions for patients without relapse. The median MTV was 652.2 mL for patients with an event within 2 years and 351.4 mL for patients without relapse. All dissemination features (number of lesions, Dmax_patient_, Dmax_bulk_, Spread_patient_, Spread_bulk_) were higher for patients with an event within 2 years (Table 3; Fig. 1). Dissemination features correlated poorly with the natural logarithm of MTV. Moreover, Dmax_bulk_ correlated poorly with the height of patients (correlation coefficient, 0.12).

**Table 3:** Descriptive statistics of conventional PET features, dissemination features, and sphericity stratified for events and non-events

Parameter	Events (*n* = 52)		Non-events (*n* = 265)	
	Median (IQR)	Range	Median (IQR)	Range
MTV (ml)	652.2 (322.6–1363.2)	13.7–5598.5	351.4 (115.9–842.1)	0.8–2827.3
SUV_max_	20.4 (15.2–27.7)	5.4–48.3	22.6 (16.8–29.3)	4.1–56.9
SUV_peak_	16.4 (12.1–21.8)	4.1–34.8	17.8 (13.8–24.3)	2.5–47.7
SUV_mean_	8.4 (6.0–9.8)	4.2–13.6	8.7 (6.9–10.6)	4.1–21.5
TLG	6030.9 (2446.1–10,571.8)	59.3–47,965.7	3216.1 (1041.5–7091.3)	0.3–25,776.8
Number of lesions	19 (6–35)	1–143	8 (4–16)	1–55
Dmax_patient_ (cm)	63.9 (43.4–70.3)	0–114.2	40.8 (15.9–58.3)	0–126.1
Dmax_bulk_ (cm)	44.4 (32.6–54.2)	0–110.8	29.4 (13.1–43.0)	0–87.0
spread_patient_ (cm)	7482.3 (734.0–37,496.1)	0–968,211	607.6 (108.0–4995.9)	0–175,968)
spread_bulk_ (cm)	604.3 (156.1–1193.0)	0–6067.6	148.1 (46.3–429.5)	0–4406.9
sphericity	0.31 (0.23–0.42)	0.13–0.68	0.39 (0.29–0.53)	0.08–1.0

Abbreviations: *MTV*, metabolic tumor volume; *SUV*, standardized uptake value; *TLG*, total lesion glycolysis; *Dmax*, maximum distance

### Performance currently used predictors

**Fig. 2:**
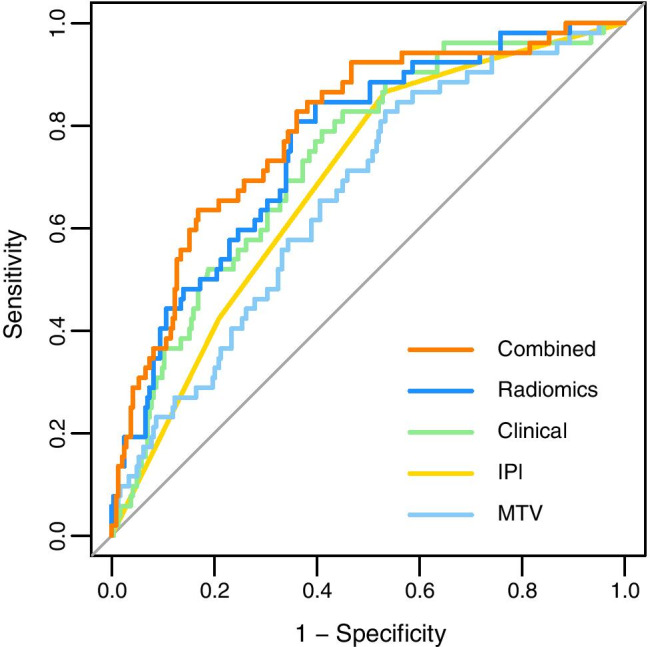
Receiver operating characteristic curves for 2-year time to progression for IPI, best clinical, MTV, best radiomics model, and combined prediction models

*IPI* (model 1) was significantly associated with outcome (*p* < 0.001), yielding an AUC of 0.68 (95% CI, 0.61–0.75) (Fig. 2, Table 4). In a multivariate logistic regression of individual IPI components and bulky disease with backward selection (model 2), the natural logarithm of LDH/ULN (*p* = 0.014), WHO performance status ≥ 1 (*p* = 0.026), and EN involvement ≥ 1 (*p* = 0.039) were all significantly associated with 2-year TTP, and together yielded an AUC of 0.73 (95% CI, 0.66–0.80). This was not significantly higher than the discriminative power of IPI (model 1) (*p* = 0.267).

**Table 4:** AUC’s, CV-AUCs, and diagnostic measures of prediction models

Model	AUC (95%CI)	CV-AUC (95%CI)	Log-likelihood ratio	Specificity	Sensitivity	NPV	PPV
IPI (model 1)	0.68 (0.61–0.75)	0.68 (0.51–0.80)	− 126.11	0.79	0.40	0.86	0.29
Clinical model (model 2)	0.73 (0.66–0.80)	0.71 (0.56–0.86)	− 123.52	0.87	0.38	0.87	0.38
MTV (model 3)	0.66 (0.58–0.74)	0.66 (0.50–0.81)	− 129.96	0.84	0.27	0.84	0.27
Limited radiomics model (model 4)	0.76 (0.69–0.82)	0.75 (0.59–0.88)	− 117.61	0.88	0.44	0.88	0.44
Combined model (model 6)	0.79 (0.73–0.86)	0.77 (0.61–0.90)	− 113.4	0.88	0.44	0.88	0.44

Abbreviations: *AUC*, area under the curve; *CV-AUC*, cross-validated AUC; *NPV*, negative predictive value; *PPV*, positive predictive value; *IPI*, international prognostic index; *MTV*, metabolic tumor volume

### Added value of radiomics features

The natural logarithm of *MTV* (model 3) was significantly associated with outcome (*p* < 0.001), yielding an AUC of 0.66 (95% CI, 0.58–0.74). The natural logarithms of MTV (*p* < 0.001) and of SUV_peak_ (*p* < 0.001) and Dmax_bulk_ (*p* = 0.001) were all significantly associated with 2-year TTP, and together yielded an AUC of 0.76 (95% CI, 0.69–0.82) for the limited radiomics model (model 4) using logistic regression with backward selection. When correcting Dmax_bulk_ for height, the radiomics model still yielded in an AUC of 0.76. This model showed a trend for better discriminative power compared to the IPI prediction model (model 1, *p* = 0.068) but was significantly higher than the discriminative power of MTV only (model 3, *p* = 0.012). LASSO regression with all radiomics features yielded a CV-AUC of 0.67 for the largest lesion and a CV-AUC of 0.54 for the hottest lesion (model 5). For both models, texture features have contributed most to the model (Supplemental data).

When currently used clinical predictors and radiomics features at patient level were combined (model 6), the natural logarithms of MTV (*p* < 0.001) and of SUV_peak_ (*p* < 0.001), Dmax_bulk_ (*p* = 0.002), WHO performance status ≥ 1 (*p* = 0.044), and age > 60 (*p* = 0.045) were all significantly associated with 2-year TTP, and together yielded an AUC of 0.79 (95% CI, 0.73–0.86) in a multivariate model. This combination showed better discriminative power compared to the IPI model (model 1, *p* = 0.003) and the best clinical prediction model (model 2, *p* = 0.049) and a trend for better discriminative power than the best radiomics prediction model (model 4, *p* = 0.051). Model performances and feature selection using LASSO regression for model 1–4 and 6 are presented in the Supplemental data (Supplemental Table 2).

For the sensitivity analysis with 2-year PFS as outcome parameter, multivariate logistic regression with backward selection resulted in selection of the same features for the radiomics and combined prediction models. For the best clinical model, extranodal involvement ≥ 1 was significantly associated with outcome and added to the prediction model. The combined model (model 6) had higher discriminative power compared to the IPI prediction model (model 1, *p* = 0.009). For all prediction models, AUCs with 2-year PFS as outcome parameter were lower compared to AUCs with 2-year TTP as outcome parameter (Supplemental Table 3).

### Diagnostic performance prediction models

Using the prevalence of progression to define the high-risk group, specificity, sensitivity, NPV, and PPV increased, and log-likelihood ratio’s decreased when adding radiomics features to currently used clinical predictors (Table 3). Sensitivity ranged between 27% for the MTV model and 44% for best radiomics and combined prediction models. Specificity was always above 79% and the highest for the radiomics and combined models (88%). The NPV was high for all models and always above 84%. The PPV was generally low (< 50%). Both PPV and NPV were highest for the best radiomics model and combined model (PPV, 44%, and NPV, 88%, respectively). Moreover, the log-likelihood ratio was lowest for the combined model.

### Survival analysis

**Fig. 3:**
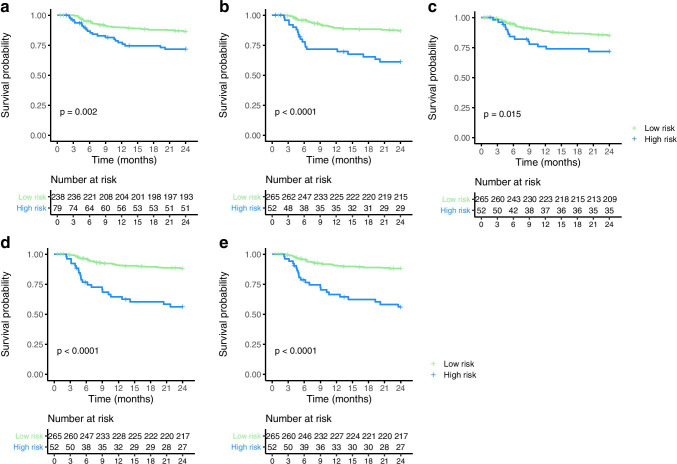
Kaplan–Meier survival curves for time to progression in months stratified by high risk and low risk according to prediction models **A** international prognostic index (IPI), prediction model, **B** clinical prediction model, **C** metabolic tumor volume (MTV) prediction model, **D** limited radiomics prediction model, and **E** combined prediction model

High-risk patients had significantly lower survival than low-risk patients for all prediction models (all, *p* < 0.015; Fig. 3, Supplemental Fig. 1). Twenty-eight percent of the high-risk patients identified by the MTV and IPI prediction models (models 1 and 3) showed progression at 2-year TTP; 40% of the high-risk clinical patient showed progression (model 2). The radiomics and combined prediction models (models 4 and model 6) correctly identified more patients; 44% of the high-risk patients showed progression at 2-year TTP. Univariate HRs for high-risk versus low-risk groups were lowest for the MTV model (HR, 2.2 (95% CI, 1.1–3.9)); HRs were higher for the IPI model (HR, 2.3 (95% CI, 1.3–4.1)), the best clinical model (HR, 3.6 (95% CI, 2.1–6.4)), and combined model (HR, 4.6 (95% CI, 2.6–7.9). Univariate HRs for high-risk versus low-risk groups were highest for the best radiomics model (HR, 4.7 (95% CI, 2.7–8.1)).

## Discussion

Results from study indicate that baseline radiomics features are predictive of outcome and have added value compared to currently used clinical parameters. Adding radiomics features can significantly increase the efficiency of clinical trials.

Currently used clinical scoring systems, such as the IPI, fail to identify a high-risk group for which novel treatment approaches are most needed [3, 4]. Combining clinical predictors and radiomics features improved model performance significantly, from an AUC of 0.68 to an AUC of 0.79. Age and WHO performance status were the only clinical predictors that remained significant. In this model, disease burden is expressed as MTV, dissemination, and intensity and combined with physical capacity to tolerate therapy, expressed as age and WHO performance status; the risk of relapse was predicted most accurately. Radiomics features had higher relative effect on the prediction of relapse compared to the clinical parameters (Supplemental data). Contrary to our results, a recent study showed that in a multivariate analysis with age-adjusted IPI (aaIPI) and radiomics feature, aaIPI was no longer a significant predictor of outcome [14], which could be caused by the smaller sample size or their choice to add aaIPI, instead of individual predictors.

The PPV increased with 15% when adding radiomics features compared the IPI model but still remained under 50%. Because of effective treatment regimens, event rates in DLBCL are low. In our database, the prior probability (i.e., the prevalence) of an event was 16%. By selecting high-risk patients with our combined prediction model, the posterior probability (i.e., PPV) of an event in this group increased to 44%. There are more high-risk patients included in the high-risk group identified using radiomics features combined with clinical parameters compared to the IPI model, as shown by higher progression rate at 2-year TTP (44% vs 28%, respectively). These survival rates are still rather high, meaning that even the best model poorly identifies real high-risk patients; this may be partly caused by our choice of outcome parameter. We chose TTP instead of the more commonly used PFS and overall survival (OS), because unlike TTP, both PFS and OS are affected by age [5]. Patients with DLBCL are generally older, and outcome of these elderly patients is not only determined by lymphoma but also by age-related comorbidities, adverse treatment effects, and limited life expectancy in general. In our dataset, 14 patients died within 2 years without signs of progression (i.e., 21.2% of PFS events). Death is a competing risk for progression. Our sensitivity analysis showed that 2-year PFS as outcome parameter showed lower predictive performance compared to 2-year TTP for all models, which could indicate that the outcome of these 14 patients is indeed unrelated to lymphoma.

Radiomics features could increase the efficiency of the design of future clinical trials for new therapies. By only selecting the high-risk patients according to our proposed prediction model, fewer patients that will not experience an event will be included. Since about 44% of the patients will experience progression, depending on the expected effectivity of the proposed drug, the difference between standard and new therapies can be studied under optimal power conditions. This allows for smaller sample sizes and thus lower costs.

MTV is one of the most studied radiomics features in DLBCL [5–9, 27]. In our study, the AUC for MTV was 0.66, which was similar to the AUC of other recent studies (range 0.64–0.66) [14, 15, 28]. These studies mainly included advanced stage DLBCL patients, making stratification more difficult and possibly explaining the relatively low, AUCs. It should be noted that these studies used different outcome parameters (PFS) and segmentation methods (41% max and 1.5 × liver SUVmax). However, the choice of segmentation method probably does not influence the predictive value of MTV [20, 29]. Schmitz et al. [5] reported an AUC of 0.78 using the same segmentation methods and outcome parameters as in the present study. Their higher AUC may be explained by the inclusion of more low-intermediate/low-risk IPI patients in their study.

Relatively few studies have investigated the predictive value of other radiomics features in DLBCL. Moreover, due to the different features that were extracted and different numbers of features extracted, it is hard to perform a direct comparison between studies. Generally speaking, our results confirm the findings of Parvez et al., who found that radiomics features of the hottest lesion have limited predictive value [30]. Aide et al. reported that the size of regions with similar intensity in the largest lesion (long-zone high grey-level emphasis) had highest accuracy and that this was the only predictor of 2-year event-free survival in a multivariate analysis [14]. In our data, 48 out of 485 radiomics features of the largest lesion predicted 2-year TTP in univariate logistic regression models after Bonferroni-correction (data not shown), and indeed, long-zone high grey-level emphasis was one of them. Our study confirms that radiomics features of the largest lesion are predictive of outcome, albeit not as predictive as radiomics features at patient level, involving all lesions. In our study, the radiomics model with preselected conventional PET features and dissemination features had higher discriminative power than the models that included all 490 radiomics features, indicating that more complex radiomics features did not have additional predictive abilities compared to simpler radiomics features.

Cottereau et al. [15] were the first and to our knowledge the only ones to investigate the predictive value of dissemination features. They reported that Dmax_patient_ and Dmax_bulk_ were significantly associated with outcome and that Dmax_patient_ was the only predictor of outcome in multivariate analysis. In our analysis, the predictive performance of Dmax_patient_ and Dmax_bulk_ was similar, but the discriminative power for Dmax_bulk_ exceeded that of Dmax_patient_, so that Dmax_patient_ was not included in our multivariate model with backward selection. We found that adding Dmax_bulk_ and SUV_peak_ to MTV significantly improved model performance (raising AUC from 0.66 to 0.76).

Risk stratification significantly improved when combining radiomics features with clinical parameters [15, 31, 32]. Baseline ^18^F-FDG PET/CTs are already part of clinical practice; therefore, radiomics features can be calculated at no additional costs. With software becoming available that easily and reliably calculate radiomics features [18, 33], adding radiomics features to clinical scoring systems should seriously be considered. Significant efforts have been made to standardize FDG scanning, including initiatives by the European Association for Nuclear Medicine Research Limited and the US Society of Nuclear Medicine [34, 35]. However, the absence of standardized methodology hampers the use of quantitative PET parameters. The optimal cut-off of MTV and other radiomics features heavily rely on segmentation method and underlying patient data. Work is in progress to solve these methodological problems.

This study is the first to investigate the predictive value of radiomics features at patient level, for the largest lesion and the hottest lesion while combining it with currently used clinical predictors, making it the most comprehensive study so far. Even though this is the largest study that examined the predictive value of radiomics features, with 18% of the patients that were included in the prediction model having progression, this study had limited power to test more complex prediction models that included more features or to make a distinction between refractory patients and relapsed patients. Another limitation of this study is that we used a single method to segment the lymphoma lesions. Due to the large heterogeneity of tracer uptake in DLBCL lesions, choosing a single segmentation method for the whole cohort could have caused suboptimal segmentation of lesions for some patients. However, literature suggests that the fixed SUV4.0 segmentation method is successful in 78% of DLBCL patients without editing and is acceptable in 98% of patients after manual editing [20]. Moreover, the majority of our patients had advanced stage disease and were classified as high-intermediate or high risk by the IPI score. The relative lack of limited stage and low-risk DLBCL patients could influence the generalizability of our results. Lastly, harmonization methods such as ComBat have shown to be definitely worthwhile to retrospectively increase uniformity in large multicenter datasets. Therefore, ComBat-based data alignment would be a very successful approach to harmonize radiomics features between centers. However, in our study, the number of included patients per center was too small to apply ComBat..

To further investigate the predictive value of radiomics features in DLBCL, these results will be validated in a large cohort of DLBCL patients treated in different clinical trials (the PETRA cohort, https://petralymphoma.org). Moreover, the combination of radiomics and genomic features could be investigated, since both have promising results, and by combining these biomarkers, the identification of high-risk DLBCL patients could be further improved.

In conclusion, prediction models combining quantitative radiomics features extracted from baseline 18F-FDG PET/CT scans with components of the IPI score significantly improved identification of patients at risk of relapse at baseline compared to the currently used IPI score. Adding radiomics features can significantly increase the efficiency of clinical trials.

## Supplementary Information

Below is the link to the electronic supplementary material.

Supplementary file1 (DOCX 142 KB)

## Data Availability

The datasets generated during and/or analyzed during the current study are available from the corresponding author on reasonable request.
