# Supplementary material for: 18F-FDG PET baseline radiomics features improve the prediction of treatment outcome in diffuse large B-cell lymphoma
Source: Eur J Nucl Med Mol Imaging. 2021 Aug 18;49(3):932–42. doi: 10.1007/s00259-021-05480-3 (PMC8803694; doi:10.1007/s00259-021-05480-3)
Supplement: Supplementary file 1 — Supplementary file1 (DOCX 142 KB) [file 259_2021_5480_MOESM1_ESM.docx]

**Supplementary information**

**Article title:** ^18^F-FDG PET baseline radiomics features improve the prediction of treatment outcome in diffuse large B-cell lymphoma

**Journal name:** European Journal of Nuclear Medicine and Molecular Imaging

**Author names**: Jakoba J. Eertink, Tim van de Brug, Sanne E. Wiegers, Gerben J.C Zwezerijnen, Elisabeth A.G. Pfaehler, Pieternella J. Lugtenburg, Bronno van der Holt, Henrica C.W. de Vet, Otto S. Hoekstra, Ronald Boellaard, Josée M. Zijlstra

**Affiliation and e-mail address of the corresponding author:** Amsterdam UMC, Vrije Universiteit Amsterdam, department of Hematology, Cancer Center Amsterdam, De Boelelaan 1117, Amsterdam, Netherlands, e-mail: [j.zijlstra@amsterdamumc.nl](mailto:j.zijlstra@amsterdamumc.nl)

**Methods**

**Difference in survival between treatment arms.**

There was no significant difference in survival between patients treated with R-CHOP and RR-CHOP (Supplemental figure 1). 2-year time to progression (TTP) for patients treated with R-CHOP was 83.2% (95% CI: 77.5%-89.3%), and the 2-year TTP survival rate for patients treated with RR-CHOP was 82.8% (95% CI: 77.0%-89.0%).

Supplemental Figure 1. Kaplan-Meier survival curves for time to progression in months stratified for R-CHOP and RR-CHOP treatment regimens.

**Calculation of radiomics features**

Radiomics features were extracted following descriptions of the Image Biomarker Standardization Initiative using RaCaT software [1, 2]. Radiomics features are sensitive to resolution, voxel size and image noise [3, 4] therefore standardization of feature values is needed to reduce the variability of radiomics features across centers. By default, all images in RaCaT are resampled to 2x2x2 voxel size using tri-linear interpolation and intensity is discretized with a fixed bin size of 0.25 SUV before feature calculation to increase the percentage of consistent features. The exact same spatial rebinning was applied to the volumes of interest followed xwith a voxelwise 50% thresholding to generate a binary tumor map after rebinning and the latter was subsequently used to extract or calculate the radiomics features from the spatially rebinned PET images. Spatial resampling to cubic voxels led to better reproducibility of radiomics features in a multicenter setting with multiple vendors and therefore multiple voxel sizes, especially for textural feature groups. All features except the morphological features benefited from resampling. Image intensity discretization with a fixed bin width of 0.25 SUV has shown to result in higher reliability of radiomics features in a multicenter setting compared to fixed bin number discretization. Moreover, discretization with fixed bin width leads to more meaningful features that can distinguish well between tumor types [5].

2D and 3D textural features were based on the following matrices with up to 8 matrix calculation methods:

- **Grey-level co-occurrence matrix (GLCM)**: counts how often combinations of voxels with specific values are distributed along one of the image directions within a certain lesion and/or MTV [6].
- **Grey- level size zone matrix (GLSZM)**: provides information on the size of homogeneous zones for each grey-level within a certain lesion and/or MTV by counting the number of groups of connected voxels with a specific grey level value and size. Voxels are connected if the neighboring voxel has the same grey level value [7].
- **Grey-level run length matrix (GLRLM)**: provides the number of pairs of grey-level values and its length of runs within a certain lesion and/or MTV [8].
- **Grey-level distance zone matrix (GLDZM)**: describes the amount of homogeneous connected areas of a certain intensity and distance to the shape border within the lesion and/or MTV by counting the number of groups of connected voxels with a specific grey level value and distance to ROI edge [9].
- **Neighboring grey-level dependence matrix (NGLDM)**: calculates the difference of grey-levels between one voxel and its neighbors within the lesion and/or MTV by capturing the coarseness of the overall texture [10].
- **Neighborhood grey-tone difference matrix (NGTDM)**: calculates the sum of grey-level differences of voxels and the average intensity of their neighboring voxels within the lesion and/or MTV [11].

Intensity features included intensity histograms (n=24), intensity-based statistics (n=18), intensity-volume histograms (n=6) and local intensity (n=2). Details on calculation of all radiomics features can be found in the supplemental materials of Zwanenburg *et al* [2]. For the radiomics analyses at patient level, all individual lesions were summed into one volume of interest. For the radiomics analyses at lesion level, radiomics features were calculated for either the largest lesion and/or hottest lesion only.

**Features in limited radiomics model**

We used the following features as commonly used features in prediction models 4 and 6:

- Metabolic tumor volume (MTV)
- Standardized uptake value (SUV)_max_: the maximum SUV of the MTV
- SUV_peak_: the maximum average SUV within a 1 mL sphere located in the MTV
- SUV_mean_: the mean SUV of the MTV
- Total lesion glycolysis: SUV_mean_*MTV
- Dissemination features: Dmax_patient_, Dmax_bulk_, Spread_patient_ and Spread_bulk_
- Sphericity: measure of the roundness of the shape of the MTV relative to a sphere

**Statistical procedures**

We tested the predictive value of 6 prediction models. For all models except model 5, multivariate logistic regression with backward selection was used to predict outcome. We started with all potential predictors in the model and every turn the predictor with the highest *p* value was excluded from the model, until all remaining predictors were significant. For model 5, Ridge logistic regression was performed after mean centering and scaling by standard deviation of all features. Features included in the prediction models are presented in Supplemental Table 1. For prediction model 4 and 6 we preselected radiomics features that are currently used in literature to predict outcome in DLBCL. All radiomics features in these models are so-called first-order radiomics features. Prior to analysis, continuous features that had a skewness > 0.5 were log-transformed using the natural logarithm.

To evaluate model performance for 2-year TTP, the receiver-operator characteristic curve was generated to calculate the area under the curve (AUC). Stratified repeated cross-validation with 5 folds and 2000 repeats was performed to yield the cross-validated AUC (CV-AUC). The differences in model performances between prediction model 1-4 and 6, expressed as AUC, were assessed with the two-sided DeLong test for two correlated ROC curves. Comparing CV-AUCs of models is a known difficulty due to the complex relations between the trained models and the inherent dependency of train-test iterations [12]. Currently, there is no valid statistical approach with sufficient power to compare the CV-AUCs of model 5 with the CV-AUCs of the other prediction models.

Supplemental Table 1. Description of included features and features included in final model per prediction model

| **Model** | **Included features** | **Included features in final model** |
| --- | --- | --- |
| Model 1: IPI | IPI (categorized into low, low-intermediate, high-intermediate and high risk) | IPI |
| Model 2: Clinical model | Ann Arbor stage: dichotomous (stage I/II vs stage III/IV) and categorical  Age: continuous and dichotomous (age ≤60 years vs age > 60 years)  WHO performance status: 2 different cut-offs: ≥1 or ≥ 2  extranodal involvement: 2 different cut-offs: ≥ 1 and >1 LDH: dichotomous (LDH > ULN vs LDH ≤ ULN) and continuous (LDH/ULN)*.  bulky disease: dichotomous (diameter < 10 cm vs diameter ≥ 10 cm) | the natural logarithm of LDH/ULN,  WHO performance status ≥1 and EN involvement ≥1 |
| Model 3: MTV | MTV* | The natural logarithm of MTV |
| Model 4:limited Radiomics model | MTV*, SUV_max_*, SUV_peak_*, SUV_mean_*, TLG*, number of lesions*, Dmax_patient_*, Dmax_bulk_*, Spread_patient_*, Spread_bulk_* and Sphericity* | The natural logarithm of MTV, the natural logarithm of SUV_peak,_ and Dmax_bulk_ |
| Model 5: All radiomics features (largest- and hottest lesion) | 485 features for the largest* and hottest* lesion | 485 features for the largest and hottest lesion |
| Model 6: Combined model | Features model 2 and model 4 | The natural logarithm of MTV, the natural logarithm of SUV_peak_, Dmax_bulk_, WHO performance status ≥1 and age>60 |

*Abbreviations: IPI: International Prognostic Index, WHO: World Health Organization, LDH: lactate dehydrogenase, ULN: upper limit of normal, MTV: metabolic tumor volume, SUV: standardized uptake value, TLG: total lesion glycolysis, Dmax: maximum distance. * continuous features*

**Definition of high-risk group**

For the IPI prediction model, patients with 4 or 5 adverse factors according to the IPI score (high-risk IPI patients) were considered as high-risk, and patients with 0-3 adverse factors were considered as low-risk. The analyses for the IPI- and MTV prediction models were univariate, all other prediction models were multivariate. For the multivariate models we did not look at the number of adverse factors, or the cut-offs of individual predictors, but at the combined predicted probability. LDH ratio, MTV, Dmax_bulk_ and SUV_peak_ were used continuously in our prediction models without any cut-off. LDH ratio, MTV and SUVpeak were log transformed using the natural logarithm. WHO performance status (≥1) and age (>60) were used dichotomous. The sum of these individual predictors, weighted by the regression coefficients, together with the intercept of the model gives the predicted probability (expressed as log odds) of an event. High-and low-risk groups were defined based on prior probability (ie prevalence) of events [13]. In our dataset 52 patients had an event and thus should have been classified as high risk. This was 18% (52/296) of the patients in our prediction models, and 16% (52/317) of the patients for our survival analyses. Only if 52 patients were classified as high-risk, PPV and NPV could be 100%.

**Relative importance of predictors in regression models**

For all multivariate prediction models, we calculated the z-scores of individual predictors to compare the relative effects of predictors that were measured on different scales. Z-scores of the features were calculated by subtracting the respective means and dividing by the respective standard deviations. The standardized features were used as predictors in logistic regression. The absolute values of the regression coefficients quantify the relative importance of the predictors. Z-scores were calculated using logistic regression with backward feature selection for the limited radiomics, clinical and combined models. To explore feature importance of model 5, where we included all radiomics features, we derived the relative importance of each feature. The sum of all coefficients was equal to 1.0. The ten most important features are presented below .

**Limited Radiomics model regression coefficient**

Intercept                                    -1.9031

MTV                 0.9946

SUV_peak_   -0.6921

Dmax_bulk_                         0.5745

**Clinical model regression coefficient**

Intercept           -1.7788

LDH/ULN   0.3850

WHO performance status ≥1 0.3791

Extranodal involvement ≥1     0.4654

**Combined model regression coefficient**

Intercept                                   -1.9796

MTV                  0.8752

SUV_peak_ -0.7110

Dmax_bulk_ 0.5585

WHO performance status ≥1     0.3615

Age > 60         0.3691

**All radiomics features largest lesion**

**Feature group Feature Feature importance**

Intensity histogram Minimum histogram gradient 0.007037

NGLDM 3D Low dependence low grey

level emphasis 0.006103

GLCM 2D inverse difference moment

normalised 0.006052

NGDTM 2D busyness 0.005875

GLDZM 2D Grey level variance 0.005806

Statistics minimum 0.005715

GLCM 3D inverse difference normalised 0.005522

GLDZM 2D Grey level non uniformity 0.005285

Morphology area density AABB 0.005201

NGTDM 3D strength 0.005156

**All radiomics features hottest lesion**

**Feature group Feature Feature importance**

Morphology area density AABB 0.,006112

NGTDM 2D busyness 0.005636

GLCM 2D joint maximum 0.005164

Morphology flatness 0.005125

Morphology elongation 0.004958

NGLDM 2D Low dependence low grey

level emphasis 0.004901

NGLDM 3D Low dependence low grey

level emphasis 0.004884

Statistics minimum 0.004646

NGTDM 3D busyness 0.004617

intensity volume volume at intensity fraction 90 0.004613

Supplemental Table 2. Model performance expressed as CV-AUC and final feature selection using logistic regression with backward feature selection and LASSO regression

|  | CV-AUC logistic regression + backward (95% CI) | Features included in final model | CV-AUC LASSO (95% CI) | Features included final model |
| --- | --- | --- | --- | --- |
| Clinical (model 2) | 0.71 (0.56-0.86) | LDH ratio, WHO performance status (≥1), extranodal involvement (≥1) | 0.70 (0.51-0.83) | Age (dichotomous and continuous), extranodal involvement (≥1 and >1), WHO performance status (≥1 and >1), Stage (dichotomous), LDH/ULN |
| Limited Radiomics (model 4) | 0.75 (0.59-0.88) | Dmax_bulk_, MTV, SUV_peak_ | 0.72 (0.57-0.86) | SUV_peak_, MTV, Dmax_bulk_, Spread_patient_, sphericity |
| Combined (model 6) | 0.77 (0.61-0.90) | Dmax_bulk_, MTV, SUV_peak_, WHO performance status (≥1), age > 60 | 0.72 (0.54-0.86) | SUV_peak_, SUV_max_, SUV_mean_, MTV, Dmax_bulk_, Spread_patient_, age > 60, extranodal involvement (≥1 and >1), WHO performance status (≥1 and >1), LDH/ULN |

*Abbreviations: IPI: international prognostic index, MTV: metabolic tumour volume, CV-AUC: cross-validated area under the curve, CI: confidence interval.*

Supplemental Table 3. AUC’s and CV-AUCs of prediction models with 2-year time to progression and 2-year progression free survival as outcome parameters.

|  | TTP | | PFS | |
| --- | --- | --- | --- | --- |
|  | AUC | CV-AUC | AUC | CV-AUC |
| IPI (model 1) | 0.68 | 0.68 | 0.67 | 0.66 |
| Clinical model (model 2) | 0.73 | 0.71 | 0.71 | 0.69 |
| MTV (model 3) | 0.66 | 0.66 | 0.64 | 0.64 |
| Radiomics model (model 4) | 0.76 | 0.75 | 0.72 | 0.70 |
| Combined model (model 6) | 0.79 | 0.77 | 0.76 | 0.73 |

*Abbreviations: TTP: time to progression, PFS: progression free survival, AUC: area under the curve, CV-AUC: cross validated AUC, IPI: international prognostic index, MTV: metabolic tumor volume*

**References**

1. Pfaehler E, Zwanenburg A, de Jong JR, Boellaard R. RaCaT: An open source and easy to use radiomics calculator tool. PLoS One. 2019;14:e0212223. doi:10.1371/journal.pone.0212223.

2. Zwanenburg A, Vallieres M, Abdalah MA, Aerts H, Andrearczyk V, Apte A, et al. The Image Biomarker Standardization Initiative: Standardized Quantitative Radiomics for High-Throughput Image-based Phenotyping. Radiology. 2020;295:328-38. doi:10.1148/radiol.2020191145.

3. Nyflot MJ, Yang F, Byrd D, Bowen SR, Sandison GA, Kinahan PE. Quantitative radiomics: impact of stochastic effects on textural feature analysis implies the need for standards. Journal of medical imaging (Bellingham, Wash). 2015;2:041002. doi:10.1117/1.Jmi.2.4.041002.

4. Pfaehler E, van Sluis J, Merema BBJ, van Ooijen P, Berendsen RCM, van Velden FHP, et al. Experimental Multicenter and Multivendor Evaluation of the Performance of PET Radiomic Features Using 3-Dimensionally Printed Phantom Inserts. J Nucl Med. 2020;61:469-76. doi:10.2967/jnumed.119.229724.

5. Orlhac F, Soussan M, Chouahnia K, Martinod E, Buvat I. 18F-FDG PET-Derived Textural Indices Reflect Tissue-Specific Uptake Pattern in Non-Small Cell Lung Cancer. PLOS ONE. 2015;10:e0145063. doi:10.1371/journal.pone.0145063.

6. Haralick RM, Shanmugam K, Dinstein I. Textural Features for Image Classification. IEEE Trans Syst Man Cybern. 1973;SMC-3:610–21. doi:<https://doi.org/10.1109/TSMC.1973.4309314>.

7. Thibault G, Fertil B, Navarro C, Pereira S, Cau P, Levy N, et al. Shape and texture indexes application to cell nuclei classification International Journal of Pattern Recognition and Artificial Intelligence. 2013;27:1357002. doi:10.1142/s0218001413570024.

8. Chu A, Sehgal CM, Greenleaf JF. Use of gray value distribution of run lengths for texture analysis. Pattern Recognit Lett. 1990;11:415-9. doi:<https://doi.org/10.1016/0167-8655(90)90112-F>.

9. Thibault G, Angulo J, Meyer F. Advanced Statistical Matrices for Texture Characterization: Application to Cell Classification. IEEE Transactions on Biomedical Engineering. 2014;61:630-7. doi:10.1109/TBME.2013.2284600.

10. Sun C, Wee WG. Neighboring gray level dependence matrix for texture classification. Computer Vision, Graphics, and Image Processing. 1983;23:341-52. doi:<https://doi.org/10.1016/0734-189X(83)90032-4>.

11. Amadasun M, King R. Textural features corresponding to textural properties. IEEE Transactions on Systems, Man, and Cybernetics. 1989;19:1264-74. doi:10.1109/21.44046.

12. Dietterich TG. Approximate statistical tests for comparing supervised classification learning algorithms. Neural Comput. 1998;10:1895–923. doi:10.1162/089976698300017197.

13. Steyerberg EW. Clinical prediction models. New York: Springer; 2009.
